# Graphene-based molecular junctions achieve nanosecond-level reaction dynamic analysis and efficient on-chip synthesis

**DOI:** 10.1093/nsr/nwaf251

**Published:** 2025-06-20

**Authors:** Jieyi Zhang, Dong Xiang, Takhee Lee

**Affiliations:** Institute of Modern Optics and Center of Single-Molecule Science, Tianjin Key Laboratory of Micro-scale Optical Information Science and Technology, Nankai University, China; Institute of Modern Optics and Center of Single-Molecule Science, Tianjin Key Laboratory of Micro-scale Optical Information Science and Technology, Nankai University, China; Department of Physics and Astronomy, Seoul National University, Republic of Korea

A fundamental understanding of reaction mechanisms at the molecular level is crucial for optimizing reaction kinetics and product yields. Conventional techniques like nuclear magnetic resonance (NMR) spectroscopy and femtosecond laser spectroscopy have provided essential insights into reaction intermediates across a wide range of timescales [1,2]. NMR enables detailed structural analysis of relatively stable species, while femtosecond spectroscopy excels at capturing ultrafast processes on the femtosecond to picosecond scale. However, capturing and characterizing transient intermediates on the nanosecond to microsecond scale remains technically demanding. In this context, single-molecule electrical detection has emerged as a complementary approach, enabling real-time monitoring of reaction intermediates with lifetimes in the nanosecond to microsecond range.

Previous work by Guo and colleagues established a pioneering single-molecule detection platform for real-time monitoring of Suzuki–Miyaura cross-coupling reactions, offering insights into molecule-scale electrical measurements [3]. Based on molecular electronics, they demonstrated how electrical signals can be utilized to track the behavior of single molecules at the nanoscale, offering a unique approach to study complex chemical reactions. However, the temporal resolution of this approach remained limited to the microsecond scale, preventing the capture of key reaction dynamics at faster timescales.

In a recent study published in *National Science Review*, Guo and co-workers enhanced their single-molecule electrical monitoring approach by designing a graphene–molecule–graphene junction with covalent anchoring, achieving a 1.8 GHz sampling rate with a 200-MHz bandwidth current amplifier, enabling accurate detection of transient intermediates with lifetimes in the order of tens of nanoseconds (Fig. 1) [4]. This represents a significant leap in molecular electronics, where the ability to integrate graphene, a 2D material known for its remarkable electrical properties, with individual molecules allows for unprecedented control and monitoring at the molecular level. The design enabled the first real-time capture of short-lived Morita–Baylis–Hillman (MBH) intermediates (e.g. Cat-MA, with a lifetime of

∼21.8 ns) [5] and provided insights into their thermodynamic properties (e.g. Cat-MA, Δ*G* = 1.54 kcal/mol at 1 V), demonstrating the potential of electronic control to influence chemical reaction rates.

**Figure 1.**
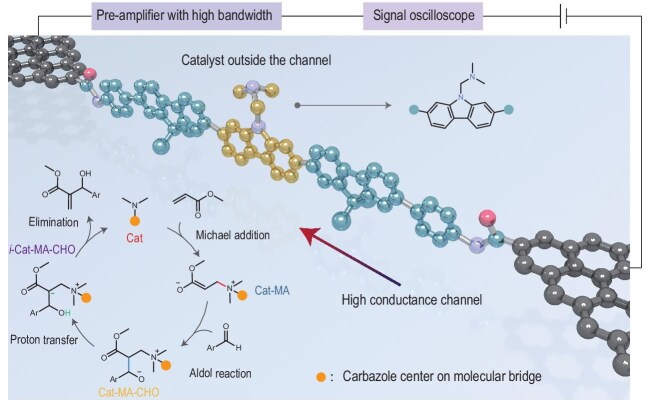
Schematic diagram of a single-catalyst device, showing electrical monitoring with nanosecond resolution. The bottom left insert shows the proposed MBH reaction mechanism. The figure is adapted from Ref. [4].

This advancement made it possible to detect hidden intermediates that were previously undetectable by conventional methods and offered deeper insights into the reaction mechanism, especially the proton transfer pathways. The two key proton transfer mechanisms, the concerted proton shuttle and stepwise acid–base processes, were identified, offering a more comprehensive understanding of the reaction dynamics and revealing how these pathways contribute to the overall catalytic process. The integration of molecular electronics with reaction monitoring provides an innovative way to study and optimize reaction conditions with high precision and temporal resolution. Furthermore, the application of a 1-V bias voltage significantly accelerated the reaction, achieving a turnover frequency of ∼5000 s⁻^1^, effectively addressing the typical challenges of slow reaction rates and low yields in MBH reactions. This demonstrates the power of molecular electronics in optimizing chemical processes, where electric field modulation can enhance reaction efficiency at the single-molecule level.

This study represents a transformative advancement in single-molecule detection technologies by achieving unprecedented nanosecond temporal resolution—an improvement of three orders of magnitude over previous microsecond-scale monitoring of Suzuki–Miyaura reactions. By integrating high-temporal-resolution monitoring with scalable single-molecule device fabrication, this work establishes a new paradigm that bridges fundamental mechanistic studies with practical applications in molecular electronics. The combination of ultrafast temporal resolution, precise electrical control and device scalability positions this technology as a powerful platform for advancing our understanding of reaction dynamics and enabling high-throughput molecular synthesis. This finding holds significant promise for pharmaceutical process development and industrial chemical manufacturing, underscoring the potential of molecular electronics to serve as an enabling technology for transformative advances in chemical catalysis and molecular synthesis.

## References

[1] Wang M, Wang T, Ojambati OS et al. Nat Rev Chem 2022; 6: 681–704. 10.1038/s41570-022-00423-4 37117494

[2] Xu X, Qi Q, Hu Q et al. Phys Rev Lett 2024; 133: 233001. 10.1103/PhysRevLett.133.233001 39714654

[3] Yang C, Zhang L, Lu C et al. Nat Nanotechnol 2021; 16: 1214–23. 10.1038/s41565-021-00959-4 34475558

[4] Yang C, Zhou S, Guo Y et al. Natl Sci Rev 2025; 172: nwaf172. 10.1093/nsr/nwaf172 PMC12342527 40809874

[5] Liu Z, Patel C, Harvey JN et al. Phys Chem Chem Phys 2017; 19: 30647–57. 10.1039/C7CP06508F 29116284

